# Supplementary material for: Mass Spectrometry-Based Proteomic Analysis of Potential Host Proteins Interacting with GP5 in PRRSV-Infected PAMs
Source: Int J Mol Sci. 2024 Feb 28;25(5):2778. doi: 10.3390/ijms25052778 (PMC10932240; doi:10.3390/ijms25052778)
Supplement: Supplementary file 1 [file ijms-25-02778-s001.zip › Table S2.pdf]

**Table S2.** KEGG pathway annotation of the cellular proteins interacting with GP5.

| No. | Pathway | Count | Pathway ID | FDR[1] |
|---|---|---|---|---|
| 1 | Metabolic pathways | 93 | ssc01100 | 0.230430931 |
| 2 | Ribosome | 37 | ssc03010 | 3.46E-13 |
| 3 | Spliceosome | 32 | ssc03040 | 6.29E-12 |
| 4 | Protein processing in endoplasmic reticulum | 32 | ssc04141 | 1.11E-08 |
| 5 | Nucleocytoplasmic transport | 27 | ssc03013 | 9.84E-10 |
| 6 | Carbon metabolism | 24 | ssc01200 | 2.01E-07 |
| 7 | Tight junction | 21 | ssc04530 | 0.003033997 |
| 8 | Biosynthesis of amino acids | 20 | ssc01230 | 6.82E-08 |
| 9 | Diabetic cardiomyopathy | 20 | ssc05415 | 0.057119462 |
| 10 | Regulation of actin cytoskeleton | 19 | ssc04810 | 0.212621723 |
| 11 | Endocytosis | 19 | ssc04144 | 0.296277328 |
| 12 | Motor proteins | 18 | ssc04814 | 0.088360264 |
| 13 | Phagosome | 16 | ssc04145 | 0.054007135 |
| 14 | Glycolysis/Gluconeogenesis | 15 | ssc00010 | 3.61E-05 |
| 15 | Autophagy - animal | 15 | ssc04140 | 0.212621723 |
| 16 | Proteasome | 14 | ssc03050 | 6.15E-06 |
| 17 | Cell cycle | 14 | ssc04110 | 0.243744964 |
| 18 | Aminoacyl-tRNA biosynthesis | 13 | ssc00970 | 3.26E-05 |
| 19 | Ribosome biogenesis in eukaryotes | 13 | ssc03008 | 0.004402597 |
| 20 | Ubiquitin mediated proteolysis | 13 | ssc04120 | 0.296277328 |
| 21 | mRNA surveillance pathway | 12 | ssc03015 | 0.053407413 |
| 22 | Pyruvate metabolism | 11 | ssc00620 | 6.05E-04 |
| 23 | Bacterial invasion of epithelial cells | 11 | ssc05100 | 0.047916385 |
| 24 | RNA degradation | 11 | ssc03018 | 0.05370587 |
| 25 | Mitophagy - animal | 11 | ssc04137 | 0.179698665 |
| 26 | Antigen processing and presentation | 10 | ssc04612 | 0.040848582 |
| 27 | Glucagon signaling pathway | 10 | ssc04922 | 0.315157991 |
| 28 | Citrate cycle (TCA cycle) | 9 | ssc00020 | 9.18E-04 |
| 29 | Fatty acid degradation | 9 | ssc00071 | 0.012646221 |
| 30 | Valine, leucine and isoleucine degradation | 9 | ssc00280 | 0.040848582 |
| 31 | Fatty acid metabolism | 9 | ssc01212 | 0.057119462 |
| 32 | Adherens junction | 9 | ssc04520 | 0.402628676 |
| 33 | N-Glycan biosynthesis | 8 | ssc00510 | 0.110512661 |
| 34 | Cysteine and methionine metabolism | 8 | ssc00270 | 0.114481982 |
| 35 | DNA replication | 7 | ssc03030 | 0.057119462 |
| 36 | Ferroptosis | 7 | ssc04216 | 0.114481982 |
| 37 | Longevity regulating pathway-multiple species | 7 | ssc04213 | 0.352758018 |
| 38 | Legionellosis | 7 | ssc05134 | 0.352758018 |
| 39 | Butanoate metabolism | 6 | ssc00650 | 0.057119462 |
| 40 | Glyoxylate and dicarboxylate metabolism | 6 | ssc00630 | 0.114481982 |
| 41 | Propanoate metabolism | 6 | ssc00640 | 0.127192037 |
| 42 | Various types of N-glycan biosynthesis | 6 | ssc00513 | 0.303807544 |
| 43 | Tryptophan metabolism | 6 | ssc00380 | 0.369677155 |
| 44 | One carbon pool by folate | 5 | ssc00670 | 0.078792464 |
| 45 | Fatty acid elongation | 5 | ssc00062 | 0.212621723 |
| 46 | 2-Oxocarboxylic acid metabolism | 5 | ssc01210 | 0.315157991 |
| 47 | Antifolate resistance | 5 | ssc01523 | 0.340499473 |
| 48 | Non-homologous end-joining | 4 | ssc03450 | 0.14870892 |

[1] FDR: false discovery rate.
