# Supplementary material for: Mass Spectrometry-Based Proteomic Analysis of Potential Host Proteins Interacting with GP5 in PRRSV-Infected PAMs
Source: Int J Mol Sci. 2024 Feb 28;25(5):2778. doi: 10.3390/ijms25052778 (PMC10932240; doi:10.3390/ijms25052778)
Supplement: Supplementary file 1 [file ijms-25-02778-s001.zip › Table S4.pdf]

Table S4. Primer sequences used in this study.

Name[1]	Sequences (5′ to 3′)[2]	GenBank	Product size
Flag-TAP1-F	ATGGAGGCCCGAATTCGGATGGCCAGCTCGGGGTC	NM_001044581	2241bp
Flag-TAP1-R	AGATCTCGGTCGACCGAATTCTCACTCAGGAGCATCTGACCCAC		
Flag-TAP2-F	ACGC*GTCGAC*CATGCGGCTCCCTGACCTG	NM_001206441	2180bp
Flag-TAP2-R	CGG*GGTACC*TCAAAGCAGGAGCTCCTCTG		
Flag-CANX-F	ACGC*GTCGAC*CATGGAAGGGAAGTGGTTGCT	NM_001243210	1782bp
Flag-CANX-R	CGG*GGTACC*TCACTCTCTTCGTGGCTTTCT		
Flag-CALR-F	CCC*AAGCTT*ATGCTGCTCCCAGTCCCACT	GQ984146	1254bp
Flag-CALR-R	ACGC*GTCGAC*CTACAGCTCATCCTTGGCCTG		
Flag-SLA-II-F	ACGC*GTCGAC*CATGTTGCATCTGTGTTTCTCC	KU754592	802bp
Flag-SLA-II-R	CGG*GGTACC*TCAGCTCAGGAGGCCTGTT		
Flag-PSMB7-F	ACGC*GTCGAC*CATGGCGGCTGTGTCAGTGTAT	DQ629163	834bp
Flag-PSMB7-R	CGG*GGTACC*TCAGGACGTGTCCATTGTCTG		
Flag-VPS16-F	CCC*AAGCTT*ATGGACTGCTACACCGCG	XM_003134328	2520bp
Flag-VPS16-R	CCG*GAATTC*TCACTTCTTTTGGGCTTGTG		
Flag-VPS18-F	CCC*AAGCTT*ATGGCGTCTATCTTGGATGAATAT	XM_001929361	2922bp
Flag-VPS18-R	CCG*GAATTC*CTACAACCAACTGAGGTGCTCC		
Myc-GP5-F	CCC*AAGCTT*AGCAACAACAGCAGCTCTCATAT	KX766378	528bp
Myc-GP5-R	CCG*CTCGAG*CTATAGACGACCCCATCGTTCC		

[1] F: forward primer, R: reverse primer.

[2] The italicized alphabets indicate restriction enzyme cleavage sites for cloning.
